# Incidence and Trends of the Leading Cancers with Elevated Incidence Among American Indian and Alaska Native Populations, 2012–2016

**DOI:** 10.1093/aje/kwaa222

**Published:** 2021-04-06

**Authors:** Stephanie C. Melkonian, Hannah K. Weir, Melissa A. Jim, Bailey Preikschat, Donald Haverkamp, Mary C. White

**Affiliations:** Division of Cancer Prevention and Control, Centers for Disease Control and Prevention, Albuquerque, New Mexico (Stephanie C. Melkonian, Melissa A. Jim, Donald Haverkamp); Division of Cancer Prevention and Control, Centers for Disease Control and Prevention, Atlanta, Georgia (Hannah K. Weir and Mary White); Institute for Integration of Medicine & Science-Community Engagement, University of Texas Health Science Center, San Antonio, Texas (Bailey Preikschat).

**Keywords:** Cancer incidence, American Indian, Alaska Native, trends, health disparity

## Abstract

Cancer incidence varies among American Indian and Alaska Native (AI/AN) populations, as well as between AI/AN and White populations. This study examined trends for cancers with elevated incidence among AI/AN compared with non-Hispanic White populations and estimated potentially avoidable incident cases among AI/AN populations. Incident cases diagnosed during 2012–2016 were identified from population-based cancer registries and linked with the Indian Health Service patient registration databases to improve racial classification of AI/AN populations. Age-adjusted rates (per 100,000) and trends were calculated for cancers with elevated incidence among AI/AN compared with non-Hispanic White populations (rate ratio >1.0), by region. Trends were estimated using joinpoint regression analyses. Expected cancers were estimated by applying age-specific cancer incidence rates among non-Hispanic White populations to population estimates for AI/AN populations. Excess cancer cases among AI/AN populations were defined as observed minus expected cases. Liver, stomach, kidney, lung, colorectal and female breast cancers had higher incidence rate among AI/AN populations across most regions. Between 2012 and 2016, nearly 5,200 excess cancers were diagnosed among AI/AN populations, with the largest number of excess cancers (1,925) occurring in the Southern Plains region. Culturally informed efforts may reduce cancer disparities associated with these and other cancers among AI/AN populations.

Previous data showed that cancer incidence rates among American Indian and Alaska Native (AI/AN) populations varied substantially from those of the general US population [1]. In addition, cancer incidence rates among AI/AN populations varied by geographic region and cancer type. Therefore, cancer incidence data, aggregated at the national level, is likely to mask substantial disparities and variations in cancer incidence rates among AI/AN populations and between AI/AN and non-Hispanic White populations.

The present study provides an overview of the leading cancer types with elevated incidence rates among AI/AN populations compared to the non-Hispanic White population during 2012–2016. We identified cancers with elevated incidence among AI/AN populations overall and by region and assessed the long-term trends of these cancers during 1999–2016. Prior studies have shown disparities in cancer incidence rates for common cancer types among AI/AN and non-Hispanic White populations; however, these studies did not specifically evaluate the cancers that disproportionately affect AI/AN populations. The purpose of this study is to highlight which cancers contribute to the largest relative disparities among AI/AN populations and to quantify the impact of these disparities (i.e., excess cases) between AI/AN and non-Hispanic White populations by geographic region. These data provide information that could be used to target public health interventions to reduce health inequities among AI/AN populations.

## METHODS

Cancer incidence data came from the National Program of Cancer Registries (NPCR) of the Centers for Disease Control and Prevention and the Surveillance, Epidemiology and End Results (SEER) Program of the National Cancer Institute [2, 3]. During the period covered by this study (2012–2016 for rate and 1999–2016 for trends), tumor histology, tumor behavior, and primary cancer site were coded according to the *Third Edition of the International Classification of Disease for Oncology (ICD-O-3) and classified according to SEER site categories* [4].

To reduce racial misclassification of AI/AN populations, cancer registry data were linked with the IHS patient registration database by using previously established and validated techniques that improve accuracy of cancer incidence estimates among the AI/AN population [5, 6]. Each year, NPCR- and SEER-funded central registries submit data on cancers diagnosed during the most recent year to the respective program.

Incidence data from registries meeting rigorous quality control standards were combined into an analytic file *U.S. Cancer Statistics* [7]. By combining these registries, we have 100% coverage of the AI/AN population. To improve racial classification of AI/AN populations, we restricted analyses to purchased/referred care delivery area (PRCDA) counties, which contain, or are adjacent to, federally recognized tribal lands. Restricting to PRCDA counties provides more accurate correction for racial misclassification of the AI/AN population than other counties [5, 6]. Approximately 53% of the AI/AN population resides in these counties (Web Figure 1).

Population estimates that are used as denominators in the rate calculations are produced by the US Census Bureau. In a previous report, the updated, bridged, intercensal population estimates overestimated AI/AN populations of Hispanic origin [8]. In the present study, all analyses were limited to non-Hispanic AI/AN populations to avoid underestimation of incidence rates among the AI/AN population. Non-Hispanic White was chosen as the reference population. For conciseness, the term “non-Hispanic” was omitted when discussing both groups in this study.

### Statistical Analysis

Cancer incidence rates for the 15 most common cancers were expressed per 100,000 population and were directly age-adjusted by using 19 age groups to the 2000 US standard population using SEER*Stat software version 8.3.2 [9]. Using the age-adjusted incidence rates, we calculated age-standardized rate-ratios (RRs) for the years 2012–2016 among the AI/AN population, with the White population as reference for each region. The leading cancer types with elevated incidence were identified for each region and sex separately. From the 15 most common cancers overall, the leading cancers with elevated incidence were identified as cancers with a rate ratio >1 (P<0.05) and ranked based on rate ratio. The six geographic regions and PRCDA counties have been described previously [1] and are shown in Web Figure 1. They include Alaska, the Northern Plains, Southern Plains, Pacific Coast, East, and Southwest. Rate ratios comparing males versus females were also calculated.

Long-term cancer incidence trends during 1999–2016 (average annual percent change, or AAPC) were estimated by joinpoint regression for the leading elevated cancers in each region. Trends for the entire period were estimated by using software developed by the NCI (Joinpoint Regression Program, Version 4.3.10) [10].

To estimate the number of excess cancers experienced among AI/AN populations, the 5-year, age-specific, cancer incidence rates among the White population were applied to the corresponding population estimates among the AI/AN populations, by sex, for the cancer types with elevated incidence in each region during 2012–2016. Excess cancers were calculated as observed minus expected cases for each cancer type, similar to previous studies that have examined elevated incidence and death [11, 12]. The observed to expected ratio was calculated to determine relative elevated incidence between the AI/AN and White populations.

## RESULTS

**Figure 1.**
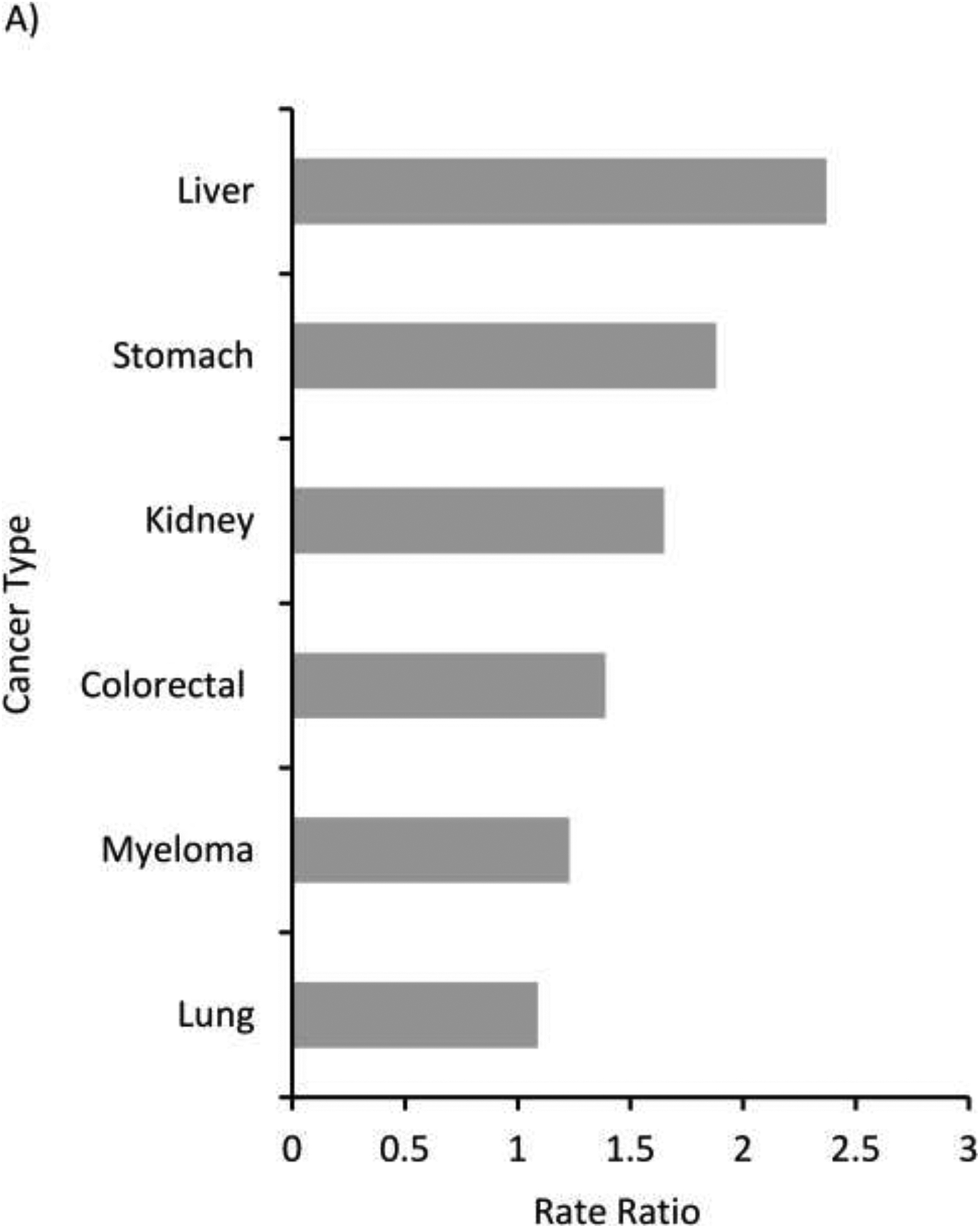

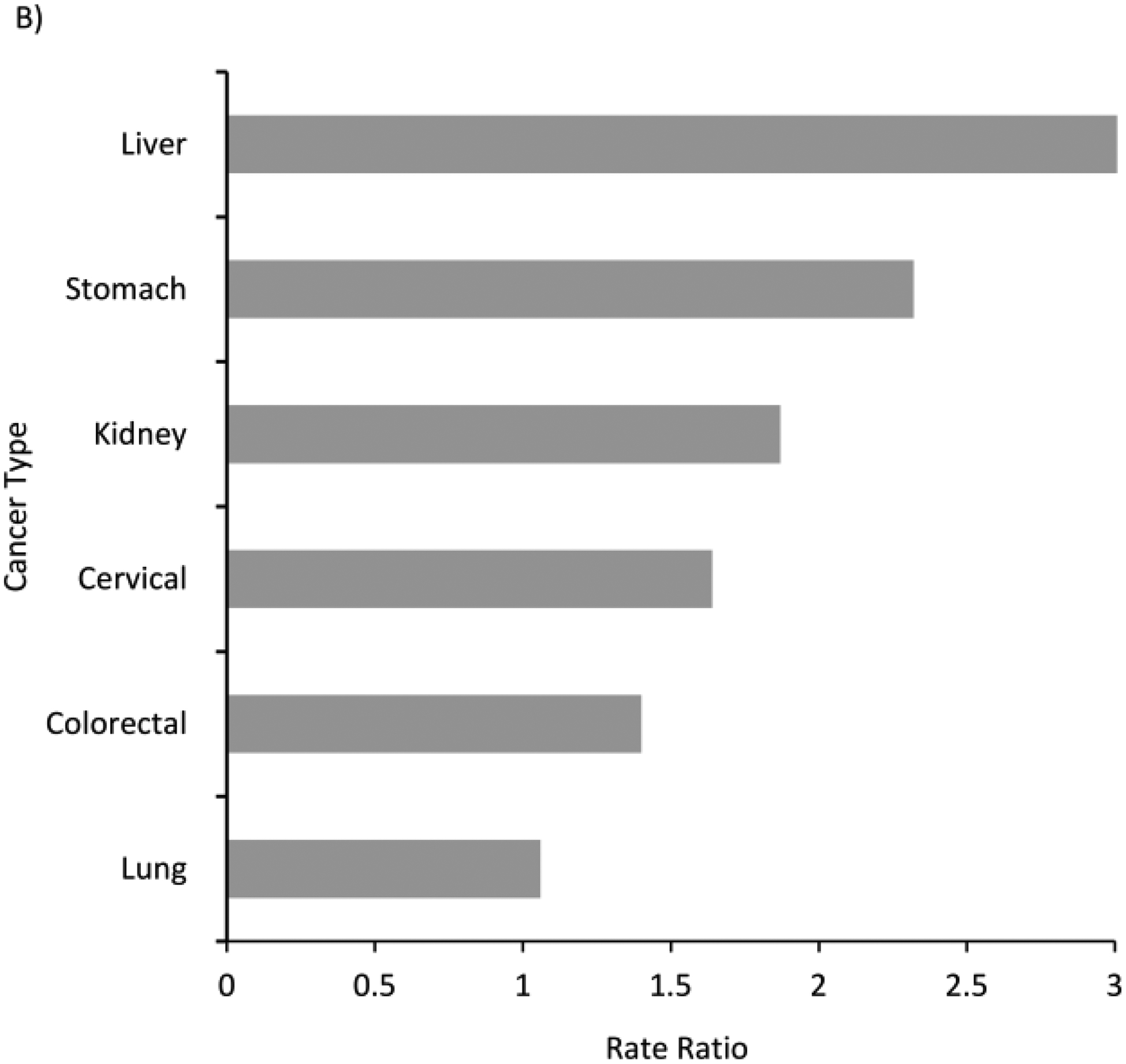
Leading cancers with elevated incidence ranked by rate ratio (AI/AN versus White) among AI/AN, Purchased/Referred Care Delivery Areas (PRCDA), overall United States, 2012–2016. A. Males, B. Females. PRCDA indicates Purchased/Referred Care Delivery Areas; AI/AN: American Indians/Alaska Natives. Includes only AI/AN of non-Hispanic origin. Leading cancers with elevated incidence selected from 15 most common cancers. See Web Tables 1 and 2. Source: Cancer registries in the Centers for Disease Control and Prevention’s National Program of Cancer Registries (NPCR) and/or the National Cancer Institute’s Surveillance, Epidemiology and End Results Program (SEER). Rate ratios (RR) are AI/AN versus White and are calculated in SEER*Stat prior to rounding of rates and may note equal the RR calculated from the rates presented in the table. All excess burden cancers shown here are significantly higher in the AI/AN versus White populations (P<0.05)

**Figure 2:**
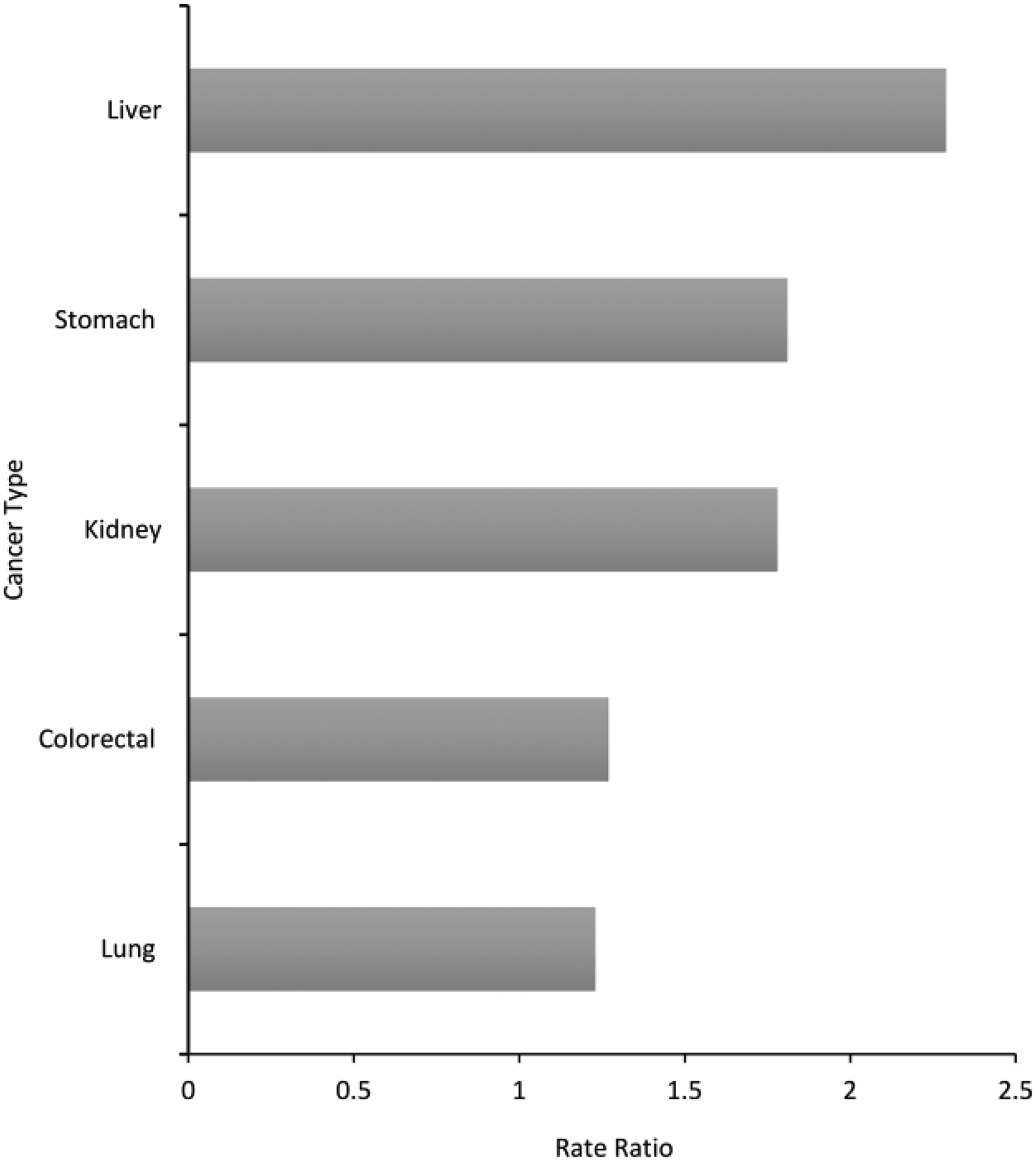
Male versus female rate ratio for leading cancers with elevated incidence among AI/AN populations, PRCDA, 2012–2016. Leading cancers with elevated incidence selected from 15 leading cancers. See Web Tables 1 and 2. Source: Cancer registries in the Centers for Disease Control and Prevention’s National Program of Cancer Registries (NPCR) and/or the National Cancer Institute’s Surveillance, Epidemiology and End Results Program (SEER). Rate ratios (RR) are AI/AN versus White and are calculated in SEER*Stat prior to rounding of rates and may not equal the RR calculated from the rates presented in the table. Only leading causes of excess cancer burden that were common between males and females, and significantly greater than the White population are shown in this graph.

Cancer incidence rates for the 15 most common cancers among AI/AN males and females compared to the White population, and their corresponding RRs, are shown in Web Tables 1 and 2. The leading cancers with elevated cancer incidence were selected on the basis of the RR comparing AI/AN versus White incidence rates by sex. Among AI/AN males, the leading cancer sites with elevated incidence were liver, stomach, kidney, colorectal, myeloma, and lung (Figure 1A, Web Table 1). The leading cancers with elevated incidence among AI/AN females included these sites, except for myeloma, and including cervical cancers (Figure 1B, Web Table 2). Rate ratios ranged from 1.09 (lung) to 2.37 (liver) among AI/AN males and 1.06 (lung) to 3.03 (liver) among AI/AN females. The incidence rates were higher among AI/AN males compared to females, ranging from 23% higher for lung cancer, to 129% higher for liver cancer (Figure 2).

Among AI/AN males, the number and type of cancers with elevated incidence varied by region (Table 1). Alaska, the Southern Plains, Southwest, and the Northern Plains had the highest number of cancer types with elevated incidence among the AI/AN population. Rate ratios for cancers with elevated incidence ranged from 1.14 (colorectal cancer in the Southwest) to 4.36 (stomach cancer in Alaska). Liver, stomach, kidney, lung, and colorectal cancers were elevated in most regions, except for lung cancer in the Southwest and liver cancer in Alaska. In the East, only liver cancer was significantly higher among AI/AN males compared to White males. Liver cancer was the leading site with elevated incidence in four of six regions.

Among AI/AN males, the incidence rate of several cancers increased during the study period (Table 1), with some of the largest increases occurring in liver cancers in the East (AAPC 8.1), Southern Plains (AAPC: 6.2), Pacific Coast (AAPC: 4.8) and Northern Plains (AAPC 4.5), and kidney cancers in the Northern Plains (AAPC: 2.9) and Southern Plains (AAPC: 3.1). Significant decreases were observed in colorectal cancer incidence rates in the Northern Plains (AAPC: −1.7) and Pacific Coast (AAPC −2.0).

Among AI/AN females, the Southern Plains, Northern Plains, Alaska, and the Pacific Coast had the most types of cancer with elevated incidence (Table 2). Rate ratios for elevated incidence cancers ranged from 1.15 (corpus and uterus in the Pacific Coast) to 4.07 (stomach in Alaska). Rate ratios for liver cancer ranged from 2.17 in the East and Alaska to 3.63 in the Southwest. Stomach cancer was elevated in every region. Kidney cancer was elevated in 5 of 6 regions (except the East); the highest RR occurred in the Northern Plains (RR = 2.12).

During 2012–2016, stomach cancer incidence rates decreased significantly among AI/AN females in the Northern Plains (AAPC −4.3) (Table 2). Incidence rates of liver cancer increased significantly in the Southern Plains (AAPC: 4.8) and Southwest (AAPC: 3.6). Rates of kidney cancer also increased significantly in the Southern Plains (AAPC: 2.7) and Southwest (AAPC: 2.1). Cancers of the pancreas (AAPC: 2.9), corpus and uterus (AAPC: 1.6), and thyroid (AAPC: 6.6) increased significantly in the Southern Plains; and stomach cancer increased in the East (AAPC: 4.4). Rates of breast cancer also increased in the Southern Plains (AAPC: 1.0).

For the leading sites with elevated incidence among AI/AN males (Table 3), the observed to expected ratios ranged from 1.14 for colorectal cancer in the Pacific Coast to 5.33 for stomach cancer in Alaska. An estimated 2,450 excess cancers were diagnosed among AI/AN males. The largest number of excess cancers among AI/AN males occurred in Southern Plains (727) and some of the highest numbers of excess cancers are attributable to colorectal, lung, and liver cancers.

Among AI/AN females, the observed to expected ratios for the leading causes of elevated cancer incidence (Table 4) ranged from 1.14 (corpus and uterus in the Pacific Coast) to 4.13 (stomach in Alaska). Overall, 2,732 excess cancers were diagnosed among AI/AN females in these 6 regions. The largest number of excess cancers among AI/AN females occurred in the Southern Plains (1198).

## DISCUSSION

This study provides a comprehensive overview of the leading cancer types with elevated incidence among the AI/AN population, by region, for the years 2012–2016. The cancers identified as excess cancers in this study are driving cancer disparities in AI/AN populations and have been shown to contribute to cancer related disparities in other underrepresented populations [13–15]. The present study confirms prior findings that showed substantial regional variation in cancer incidence rates among the AI/AN population [1, 16]. These findings provide further evidence that data aggregated across cancer sites or by region mask important differences in cancer incidence, both within the AI/AN population and between the AI/AN and White population. Liver, stomach, kidney, lung, and colorectal cancers were among the cancers consistently elevated across several regions among the AI/AN population. This study estimated that nearly 5,200 cases of cancer in the AI/AN population were potentially avoidable during 2012–2016 among AI/AN populations in the 6 study areas if racial disparities had not existed in cancer incidence.

Liver cancer was a leading cancer with elevated incidence among AI/AN populations in nearly every region. Incidence rates were as much as four times higher than rates among the White population among both AI/AN males and females, confirming previous findings [17]. The prevalence of hepatitis C virus (HCV) infection, a known risk factor of liver cancer, is higher among the AI/AN population than among the non-Hispanic White population [17, 18]. To increase screening for HCV among the AI/AN population in the Southern Plains, the Cherokee Nation Health Services implemented a clinical decision support tool within the health records for primary care physicians, targeting individuals at high risk of liver cancer. This effort resulted in a nearly 14% increase in the number of eligible individuals receiving HCV screening during 2012–2015, and nearly 90% of the individuals subsequently treated after HCV diagnosis achieved a cure [19]. Other efforts include collaborations between IHS and Project ECHO (Extension for Community Healthcare Outcomes) aimed at improving HCV-related care for the AI/AN population through teleconsulting and “telementoring” partnerships between specialists and providers in rural and underserved communities [20].

This study also confirmed previous findings about the elevated incidence of kidney cancer among the AI/AN population [21]. Increases in kidney cancer incidence have been linked with rising rates of obesity [21–23]. Previous studies suggested that smoking and hypertension may also play roles in elevated kidney cancer rates [24]. These few known risk factors for kidney cancer are unlikely to fully explain the observed geographic variation in kidney cancer incidence, or the elevated incidence rates among the AI/AN population. Additional studies can help better understand the factors driving differences in kidney cancer incidence between the AI/AN and White populations.

Despite ongoing efforts to reduce the high prevalence of commercial tobacco use among AI/AN populations, the present study suggests that lung cancer incidence rates have largely remained stagnant among the AI/AN population. A new Government Performance and Results Act measure was established in 2006 to track tobacco cessation service delivery among current smokers within the IHS and tribal programs [25]. Although this measure has progressively improved each year, from the baseline of 12% in 2006 to over 50% in 2016 [25], the present study suggests that expanding culturally competent tobacco control strategies for the use of commercial tobacco remains an important aspect of cancer prevention among the AI/AN population.

This study confirms persistent disparities in colorectal cancer incidence rates among the AI/AN population. Although decreasing colorectal cancer incidence rates were observed among AI/AN males in the Northern Plains and Pacific Coast regions, rates actually increased among AI/AN males in the Southwest region. There were no significant declines in colorectal cancer incidence rates among AI/AN females in regions with elevated colorectal cancer incidence, suggesting the need to increase screening for this disease. In addition, inequities in colorectal cancer risk factors could be addressed, such as higher diabetes prevalence, lower dietary intake of fruits and vegetables, and higher consumption of sugar sweetened beverages, alcohol, and tobacco products. Collaborative efforts to increase colorectal cancer screening in regions with the highest incidence of disease show promise, but alone they may not be sufficient to eliminate disparities in incidence [26–29].

Although stomach cancer incidence rates for the overall US population are low, rates of stomach cancer among the AI/AN population are high, specifically among the Alaska Native population. The association between stomach cancer and *Helicobactor Pylori* infection can likely account for a large portion of these increased rates of stomach cancer, specifically in Alaska where the prevalence of infection among the AI/AN population can range from 64% to 81%, and reinfection rates after treatment are as high as 16% [30, 31]. Although *Helicobactor Pylori* is an important risk factor, infection is not a sufficient cause of stomach cancer [32]. Other environmental and behavioral factors linked with the development of stomach cancer include high intake of salt, nitrites, and nitrates; family history; smoking; and obesity [33–36]. Screening and early detection of stomach cancer may be beneficial to high-risk AI/AN individuals, such as first-degree relatives of persons with stomach cancer, because survival rates after treatment of advanced stage disease are poor [37]. Although screening high-risk populations for stomach cancer is appropriate among countries with relatively high incidence rates, screening is generally thought to be costly and unwarranted because of the low overall burden among countries with low rates of stomach cancer, such as the United States [32]. Studies have shown that data on exposure to risk factors associated with stomach cancer can aid in the identification of high-risk subgroups for more targeted screening and intervention [38, 39]; these strategies might also be effective in Indian country [37].

This study has limitations. To reduce racial misclassification of AI/AN populations, the IHS patient registration database was linked with data from the central cancer registries. However, these linkages only address the racial misclassification for individuals that have previously accessed services through the IHS; thus, AI/AN individuals who are not members of federally recognized tribes are not included. In addition, individuals living in urban, non-PRCDA areas are not represented in these data. Results based on these data may not be generalizable to all AI/AN individuals in the United States. Finally, restriction of the analyses to non-Hispanic AI/AN populations may not accurately represent all AI/AN populations. Although this exclusion reduced overall AI/AN incidence rates by less than 5%, this exclusion may disproportionally affect cancer incidence rates in certain regions. Finally, this study uses data from central cancer registries and does not consider social determinants of health that may affect cancer incidence.

The present study highlights cancers with elevated incidence among the AI/AN compared to the White populations. Elevated incidence in liver, stomach, kidney, lung, and colorectal cancers represent important health inequities between AI/AN and White populations. Areas for improvement for cancer prevention and control among the AI/AN population include efforts to promote healthy environments and address the underlying social determinants of cancer risk. Culturally informed, community-based interventions to support healthy behaviors, reduce exposure to carcinogens, promote recommended screening for cancer or its risk factors, and increase access to preventive health services may reduce these persistent disparities in cancer incidence among the AI/AN population.

## Supplementary Material

web docs

## Figures and Tables

**Table 1:** Age-adjusted Rates and Average Annual Percent Change of Leading Cancers with Elevated Incidence^a^ for American Indians/Alaska Natives^b^ compared to Whites, By Region, Males, United States, PRCDA Counties, 2012–2016

Region	AI/AN Rate	AI/AN Count	White Rate	White Count	Rate Ratio^c^	95% CI for Rate Ratio^d^	AI/AN AAPC^f^	White AAPC^f^
**Northern Plains**								
Liver	27.5	142	8.3	2,193	3.32	2.72–4.00	4.5^g^	3.8^g^
Stomach	16.5	73	7.4	1,821	2.25	1.70–2.91	−1.2	−1.2
Kidney	45.9	238	22.6	5,524	2.03	1.76–2.34	2.9^g^	2.0^g^
Colorectal	74.9	358	42.1	10,165	1.78	1.57–2.00	−1.7^g^	−2.7^g^
Lung	109.3	457	66.9	16,838	1.63	1.47–1.81	−1.3	−1.7^g^
**Alaska**								
Stomach	28.1	64	6.4	79	4.36	3.00–6.32	−1.8	−0.8
Colorectal	90.1	180	36.5	466	2.47	2.03–2.99	−1.6	−3.9^g^
Esophageal	15.8	31	7.7	100	2.06	1.26–3.24	NA	−1.2
Lung	104.9	195	58.9	692	1.78	1.49–2.12	−0.9	−2.9^g^
Oropharyngeal	25.7	61	15.1	229	1.70	1.22–2.33	−1	−0.5
Pancreas	19.5	38	12.1	149	1.61	1.05–2.39	1.2	1.7
Kidney	29.5	69	20.9	281	1.41	1.05–1.88	0.9	0.7
**Southern Plains**								
Liver	25.7	198	10.5	1,027	2.45	2.08–2.88	6.2^g^	4.0^g^
Kidney	46.5	342	23.8	2,133	1.96	1.73–2.21	3.1^g^	2.3^g^
Stomach	12.0	75	6.9	628	1.74	1.32–2.24	−1.0	−0.1
Myeloma	12.3	77	7.5	692	1.63	1.24–2.10	2.4	1.6^g^
Colorectal	75.7	520	46.7	4,193	1.62	1.47–1.79	−0.9	−2.4^g^
Lung	112.0	730	82.5	7,719	1.36	1.25–1.47	−0.2	−2.2^g^
Esophageal	10.6	76	8	753	1.33	1.02–1.71	1.4	0.2
**Pacific Coast**								
Liver	29.5	234	11.4	6,226	2.59	2.25–2.98	4.8^g^	3.8^g^
Kidney	25.7	184	21.1	10,349	1.22	1.04–1.42	1.2	2.1^g^
Lung	69.8	431	59	29,260	1.18	1.06–1.31	−0.5	−2.8^g^
Colorectal	46.6	296	40	19,339	1.16	1.02–1.32	−2.0^g^	−2.8^g^
**East**								
Liver	19.4	60	11.3	6,216	1.72	1.29–2.25	8.1^g^	3.5^g^
**Southwest**								
Stomach	15.5	128	5.8	1,430	2.66	2.18–3.21	−1.2	−1.6^g^
Liver	22.4	221	8.7	2,251	2.58	2.22–2.98	3.9^g^	2.5^g^
Kidney	36.5	346	18.9	4,523	1.93	1.72–2.17	1.7^g^	1.9^g^
Myeloma	9.5	80	5.8	1,450	1.63	1.26–2.06	1.1	0.5
Colorectal	43.4	401	38.1	9,098	1.14	1.02–1.27	2.2^g^	−3.0^g^

PRCDA indicates Purchased/Referred Care Delivery Areas; AI/AN: American Indians/Alaska Natives; W: non-Hispanic White; RR: Rate Ratio, NA; Not Applicable, indicates trend could not be calculated.

a Leading cancers with elevated incidence selected from 15 most common cancers. See Web Table 1. Source: Cancer registries in the Centers for Disease Control and Prevention’s National Program of Cancer Registries (NPCR) and/or the National Cancer Institute’s Surveillance, Epidemiology and End Results Program (SEER).

b AI/AN race is reported by NPCR and SEER registries or through linkage with the IHS patient registration database. Includes only AI/AN of non-Hispanic origin.

c Rates are per 100,000 persons and are age-adjusted to the 2000 U.S. standard population (19 age groups - Census P25–1130).

d Rate ratios (RR) are AI/AN versus White and are calculated in SEER*Stat prior to rounding of rates and may not equal RR calculated from rates presented in table.

e Indicates significant RR, P<0.05

f AAPC: Average Annual Percent Change. Trends estimated for the years 1999–2016, calculated using joinpoint regression analysis

g Indicates significant AAPC, P<0.05

**Table 2:** Age-adjusted Rates and Average Annual Percent Change of Leading Cancers with Elevated Incidence^a^ for American Indians/Alaska Natives^b^ compared to Whites, By Region, Females, United States, PRCDA Counties, 2012–2016

Region	AI/AN Rate	AI/AN Count	White Rate	White Count	Rate Ratio^e^	95% CI for Rate Ratio	AI/AN AAPC^f^	White AAPC^f^
**Northern Plains**								
Liver	10.5	64	3.2	912	3.27	2.45–4.28	2.4	2.5^g^
Kidney	23.5	143	11.1	2,941	2.12	1.76–2.53	0.7	1.4^g^
Lung	102.0	551	53.3	15,266	1.92	1.75–2.09	0.3	−0.3
Stomach	5.9	33	3.1	863	1.92	1.29–2.74	−4.3^g^	−0.9
Cervical	12.0	73	6.3	1,294	1.90	1.47–2.42	−0.5	−1.6^g^
Colorectal	51.3	299	33	9,157	1.55	1.37–1.75	−1.5	−2.6^g^
Oropharyngeal	10.1	58	6.7	1,834	1.51	1.12–1.98	2.5	0.4
**Alaska**								
Stomach	14.5	33	3.6	43	4.07	2.44–6.71	−3.4	−3.0
Colorectal	96.3	216	32.6	376	2.96	2.47–3.53	−1.3	−2.1^g^
Oropharyngeal	13.4	32	5.1	63	2.61	1.61–4.13	−0.9	−0.3
Liver	7.7	19	3.5	45	2.17	1.15–3.88	1.4	2
Kidney	21.3	54	11.9	145	1.79	1.26–2.49	2.1	2.1
Cervical	10.9	26	6.8	79	1.60	1.00–2.56	1.1	−0.5
Lung	71.9	164	47.8	546	1.51	1.25–1.81	−0.2	−2.3^g^
Female Breast	151.0	373.0	121.0	1488	1.25	1.11–1.41	0.3	−1.3^g^
**Southern Plains**								
Liver	10.3	88	3.5	381	2.93^e^	2.27–3.73	4.8^g^	2.2^g^
Stomach	6.8	56	3.0	319	2.27^e^	1.66–3.05	−1.5	−0.7
Kidney	27.7	235	13.2	1,335	2.10^e^	1.81–2.42	2.7^g^	2.9^g^
Cervical	13.8	118.0	8.5	644	1.63	1.32–2.00	−0.8	−0.7
Pancreas	16.3	129.0	10.2	1,112	1.60	1.31–1.93	2.9^g^	1.4^g^
Colorectal	55.7	452.0	35.3	3,683	1.58	1.42–1.75	−0.4	−1.8^g^
Lung	84.6	697.0	56.6	6,224	1.49	1.38–1.62	−0.1	−0.8^g^
Corpus and Uterus, NOS	29.9	261.0	21.9	2,265	1.37	1.19–1.56	1.6^g^	0.9^g^
Female Breast	158.7	1337.0	119	11,905	1.33	1.26–1.41	1.0^g^	−0.4
Ovary	15.5	132.0	11.6	1,167	1.33	1.10–1.61	−0.5	−1.5^g^
Non-Hodgkin Lymphoma	19.9	158.0	15.1	1,582	1.32	1.11–1.56	−1.0	0.1
Thyroid	23.6	207.0	20.1	1,636	1.18	1.01–1.36	6.6^g^	5.0^g^
**Pacific Coast**								
Liver	11.2	94	3.9	2284	2.85	2.26–3.55	3.7	3.2^g^
Stomach	6.1	43	3.0	1687	2.03	1.44–2.78	1.2	−1.2^g^
Cervical	13.8	105	6.9	2,802	2.00	1.62–2.44	1.8	−0.9^g^
Kidney	16.5	134	10.4	5,566	1.59	1.32–1.90	1.5	1.9^g^
Colorectal	43.2	317	32	17,641	1.35	1.20–1.51	−0.6	−2.2^g^
Lung	60.7	456	51.5	29,686	1.18	1.07–1.30	−0.2	−1.6^g^
Corpus and Uterus, NOS	30.8	262	26.7	15,011	1.15	1.01–1.31	1.8	−0.9^g^
**East**								
Liver	7.4	22	3.4	2,138	2.17	1.33–3.35	NA	−2.5
Stomach	8.2	25	4.0	2,435	2.05	1.30–3.07	4.4^g^	−0.7
**Southwest**								
Liver	12.1	138	3.3	918	3.63	3.00–4.37	3.6^g^	2.9^g^
Stomach	8.5	93	2.7	717	3.16	2.50–3.94	−0.9	−0.8^g^
Kidney	17.2	208	9.3	2,378	1.84	1.58–2.13	2.1^g^	1.2^g^
Corpus and Uterus, NOS	28.2	353	22.5	5,912	1.25	1.12–1.40	−1.7	−1.1^g^

PRCDA indicates Purchased/Referred Care Delivery Areas; AI/AN: American Indians/Alaska Natives; W: non-Hispanic White; LCL: lower confidence level; UCL: upper confidence level; NA: Not Applicable, indicates trend could not be calculated; NOS: Not otherwise specified

a Leading cancers with elevated incidence selected from 15 most common cancers. See Web Table 2. Source: Cancer registries in the Centers for Disease Control and Prevention’s National Program of Cancer Registries (NPCR) and/or the National Cancer Institute’s Surveillance, Epidemiology and End Results Program (SEER).

b AI/AN race is reported by NPCR and SEER registries or through linkage with the IHS patient registration database. Includes only AI/AN of non-Hispanic origin.

c Rates are per 100,000 persons and are age-adjusted to the 2000 U.S. standard population (19 age groups - Census P25–1130).

d Rate ratios (RR) are AI/AN versus White and are calculated in SEER*Stat prior to rounding of rates and may not equal RR calculated from rates presented in table.

e Indicates significant RR, P<0.05

f AAPC: Average Annual Percent Change. Trends estimated for the years 1999–2016, calculated using joinpoint regression analysis

g Indicates significant AAPC, P<0.05

**Table 3:** Expected and Observed Number of Cancers and Observed to Expected Ratio for Leading Cancers with Elevated Incidence^b^ by Region, AI/AN Males, PRCDA Counties 2012–2016

	Observed Cases^c^	Expected Cases^d^	Excess Cancers	Observed to Expected Ratio
**Northern Plains**				
Liver	142	42	100	3.38
Stomach	73	32	41	2.28
Kidney	238	112	126	2.13
Colorectal	358	192	166	1.86
Lung	457	286	171	1.60
*Total Excess Cancer Cases*			*604*	
**Alaska**				
Stomach	64	12	52	5.33
Colorectal	180	74	106	2.43
Esophageal	31	15	16	2.07
Lung	195	107	88	1.82
Oral Cavity and Pharynx	61	36	25	1.69
Pancreas	38	23	15	1.65
Kidney	69	45	24	1.53
*Total Excess Cancer Cases*			*326*	
**Southern Plains**				
Liver	138	40	98	3.45
Kidney	342	174	168	1.97
Stomach	75	46	29	1.63
Myeloma	77	48	29	1.60
Colorectal	520	321	199	1.62
Lung	730	544	186	1.34
Esophageal	76	58	18	1.31
*Total Excess Cancer Cases*			*727*	
**Pacific Coast**				
Liver	234	87	147	2.69
Kidney	184	144	40	1.28
Lung	431	358	73	1.20
Colorectal	296	259	37	1.14
*Total Excess Cancer Cases*			*297*	
**East**				
Liver	60	32	28	1.88
*Total Excess Cancer Cases*			*28*	
**Southwest**				
Stomach	128	50	78	2.56
Liver	221	87	134	2.54
Kidney	346	179	167	1.93
Myeloma	80	50	30	1.60
Colorectal	401	342	59	1.17
*Total Excess Cancer Cases*			*468*	

a AI/AN race is reported by NPCR and SEER registries or through linkage with the IHS patient registration database. Includes only AI/AN of non-Hispanic origin. Source: Cancer registries in the Centers for Disease Control and Prevention’s National Program of Cancer Registries (NPCR) and/or the National Cancer Institute’s Surveillance, Epidemiology and End Results Program (SEER)

b Leading cancers with elevated incidence selected from 15 most common cancers. See Web Table 1

c Observed counts are the number of cancer cases observed in the dataset

d Expected counts are calculated by applying age specific cancer incidence rates from the White population to the AI/AN population

**Table 4:** Expected and Observed Number of Cancers and Observed to Expected Ratio for Leading Cancers with Elevated Incidence^b^ by Region AI/AN Females, PRCDA Counties 20122016

	Observed Cases^c^	Expected Cases^d^	Excess Cancers	Observed to Expected Ratio
**Northern Plains**				
Liver	64	18	46	3.56
Kidney	143	66	77	2.17
Lung	551	296	255	1.86
Stomach	33	17	16	1.94
Cervical	73	40	33	1.83
Colorectal	299	177	122	1.69
Oropharyngeal	58	40	18	1.45
*Total Excess Cancer Cases*			*567*	
**Alaska**				
Stomach	33	8	25	4.13
Colorectal	216	74	142	2.92
Oropharyngeal	32	12	20	2.67
Liver	19	9	10	2.11
Kidney	54	28	26	1.93
Cervical	26	16	10	1.63
Lung	164	105	59	1.56
*Total Excess Cancer Cases*			*292*	
**Southern Plains**				
Liver	88	30	58	2.93
Stomach	56	24	32	2.33
Kidney	235	114	121	2.06
Cervical	118	71	47	1.66
Pancreas	129	81	48	1.59
Colorectal	452	286	166	1.58
Lung	697	467	230	1.49
Corpus and Uterus, NOS	261	190	71	1.37
Female Breast	1337	1012	325	1.32
Ovary	132	99	33	1.33
Non-Hodgkin Lymphoma	I58	122	36	1.30
Thyroid	207	176	31	1.18
*Total Excess Cancer Cases*			*1198*	
**Pacific Coast**				
Liver	94	32	62	2.94
Stomach	43	22	21	1.95
Cervical	105	52	53	2.02
Colorectal	317	235	82	1.35
Lung	456	377	79	1.21
Corpus and Uterus, NOS	253	222	31	1.14
*Total Excess Cancer Cases*			*328*	
**East**				
Liver	22	11	11	2.00
Stomach	25	12	13	2.08
*Total Excess Cancer Cases*			*24*	
**Southwest**				
Liver	138	40	98	3.45
Stomach	93	30	63	3.10
Kidney	208	111	97	1.87
Corpus and Uterus, NOS	337	272	65	1.24
*Total Excess Cancer Cases*			*323*	

a AI/AN race is reported by NPCR and SEER registries or through linkage with the IHS patient registration database. Includes only AI/AN of non-Hispanic origin. Source: Cancer registries in the Centers for Disease Control and Prevention’s National Program of Cancer Registries (NPCR) and/or the National Cancer Institute’s Surveillance, Epidemiology and End Results Program (SEER)

b Leading cancers with elevated incidence selected from 15 most common cancers. See Web Table 2

c Observed counts are the number of cancer cases observed in the dataset

d Expected counts are calculated by applying age specific cancer incidence rates from the White population to the AI/AN population

## Abbreviations:

AI/AN: American Indian and Alaska Native
IHS: Indian Health Service
AAPC: Average Annual Percent Change
HCV: hepatitis C virus
PRCDA: Purchased/Referred Care Delivery Area
