# Supplementary material for: Incidence and Trends of the Leading Cancers with Elevated Incidence Among American Indian and Alaska Native Populations, 2012–2016
Source: Am J Epidemiol. Author manuscript; Available in PMC 2022 Apr 6. (PMC8026484; doi:10.1093/aje/kwaa222)
Supplement: web docs [file NIHMS1653812-supplement-web_docs.pdf]

Incidence and Trends of the Leading Cancers with Elevated Incidence Among American Indian and Alaska Native Populations, 2012–2016

Stephanie C. Melkonian, Hannah K. Weir, Melissa A. Jim, Bailey Preikschat, Donald Haverkamp and Mary C. White

Web Tables 1 and 2
Web Figure 1

Web Table 1. Leading Cancer Sites for American Indians/Alaska Natives[a] compared to Whites for the United States, Males, PRCDA Counties, US, 2012-2016

	Overall US			Northern Plains			Alaska			Southern Plains			Pacific Coast			East			Southwest		
Site (In order based on overall US rank in AI/AN)	Rank[b], AI/AN (W)	AI/AN (W) Rate[c]	RR[d]	Rank[b], AI/AN (W)	AI/AN (W) Rate[c]	RR[d]	Rank[b], AI/AN (W)	AI/AN (W) Rate[c]	RR[d]	Rank[b], AI/AN (W)	AI/AN (W) Rate[c]	RR[d]	Rank[b], AI/AN (W)	AI/AN (W) Rate[c]	RR[d]	Rank[b], AI/AN (W)	AI/AN (W) Rate[c]	RR[d]	Rank[b], AI/AN (W)	AI/AN (W) Rate[c]	RR[d]
All Cancers		472.5 (486.0)	0.97[e]		594.6 (475.5)	1.25[e]		543.9 (417.1)	1.30[e]		631.2 (490.9)	1.29[e]		459.8 (479.4)	0.96[e]		337.5 (527.8)	0.64[e]		322.3 (424.9)	0.76[e]
Prostate	1 (1)	79.2 (96.6)	0.82[e]	2(1)	105.3 (97.8)	1.08	3(1)	61.2 (83.9)	0.73[e]	2(1)	103.3 (90.0)	1.15[e]	1(1)	77.6 (95.0)	0.82[e]	1(1)	68.0 (106.3)	0.64[e]	1(1)	55.4 (82.1)	0.67[e]
Lung	2(2)	71.3 (65.6)	1.09[e]	1(2)	109.3 (66.9)	1.63[e]	1(2)	104.9 (58.9)	1.78[e]	1(2)	112.0 (82.5)	1.36[e]	2(2)	69.8 (59.0)	1.18[e]	2(2)	50.3 (73.6)	0.68[e]	5(2)	21.6 (54.7)	0.39[e]
Colorectal	3(3)	57.6 (41.3)	1.39[e]	3(3)	74.9 (42.1)	1.78[e]	2(4)	90.1 (36.5)	2.47[e]	3(3)	75.7 (46.7)	1.62[e]	3(4)	46.6 (40.0)	1.16[e]	3(4)	33.0 (42.9)	0.77[e]	2(3)	43.4 (38.1)	1.14[e]
Kidney	4(7)	36.3 (22)	1.65[e]	4(7)	45.9 (22.6)	2.03[e]	4(6)	29.5 (20.9)	1.41[e]	4(6)	46.5 (23.8)	1.96[e]	6(7)	25.7 (21.1)	1.22[e]	4(7)	21.9 (23.6)	0.92	3(7)	36.5 (18.9)	1.93[e]
Liver	5(11)	24.7 (10.4)	2.37[e]	5(12)	27.5 (8.3)	3.32[e]	12(11)	15.3 (11.4)	1.34	7(11)	25.7 (10.5)	2.45[e]	4(11)	29.5 (11.4)	2.59[e]	5(11)	19.4 (11.3)	1.72[e]	4(11)	22.4 (8.7)	2.58[e]
Urinary Bladder	6(4)	22.1 (39)	0.57[e]	6(4)	24.0 (37.7)	0.64[e]	7(3)	22.2 (37.1)	0.60[e]	5(4)	36.0 (36.0)	1.00	5(5)	26.5 (38.1)	0.70[e]	7(3)	16.2 (43.1)	0.38[e]	11(4)	8.9 (35.0)	0.26[e]
Non-Hodgkin Lymphoma	7(6)	18.2 (23.3)	0.78[e]	8(6)	20.2 (23.4)	0.86	10(7)	18.2 (20.9)	0.87	6(7)	26.1 (22.0)	1.19	7(6)	21.2 (23.5)	0.90	10(6)	9.5 (25.3)	0.38[e]	8(6)	11.7 (19.3)	0.61[e]
Oropharyngeal	8(8)	17.5 (18.8)	0.93	7(9)	21.0 (17.1)	1.23	6(8)	25.7 (15.1)	1.70[e]	8(8)	23.8 (20.4)	1.17	8(8)	18.1 (19.6)	0.92	6(9)	17.4 (19.8)	0.88	12(8)	8.5 (16.2)	0.52[e]
Leukemias	9(9)	15.5 (18.6)	0.83[e]	9(8)	18.2 (18.8)	0.96	9(9)	18.3 (12.3)	1.49	10(9)	20.3 (17.8)	1.14	10(9)	15.8 (18.2)	0.87	8(8)	15.1 (20.5)	0.74	9(9)	9.6 (15.8)	0.61[e]
Pancreas	10(10)	14.8 (14.5)	1.02	10(10)	16.6 (14.5)	1.15	8(10)	19.5 (12.1)	1.61[e]	11(10)	15.5 (12.9)	1.20	9(10)	16.9 (14.2)	1.19	9(10)	10.1 (15.6)	0.65[e]	7(10)	11.8 (13.3)	0.89
Stomach	11(16)	13.9 (7.4)	1.88[e]	11(15)	16.5 (7.4)	2.25[e]	5(15)	28.1 (6.4)	4.36[e]	13(16)	12.0 (6.9)	1.74[e]	12(15)	9.4 (7.3)	1.29	11(15)	8.4 (8.3)	1.01	6(17)	15.5 (5.8)	2.66[e]
Melanomas of the Skin	12(5)	11.2 (35.5)	0.32[e]	12(5)	11.2 (27.7)	0.40[e]	18(5)	5.7 (21.4)	0.26[e]	9(5)	21.4 (31.2)	0.69[e]	11(3)	11.6 (40.6)	0.29[e]	13(5)	6.1 (36.4)	0.17[e]	15(5)	6.0 (33.6)	0.18[e]
Myeloma	13(15)	9.2 (7.4)	1.23[e]	15(14)	10.7 (7.9)	1.35	15(17)	6.7 (5.1)	1.32	12(14)	12.3 (7.5)	1.63[e]	16(16)	6.6 (7.2)	0.92	14(16)	5.4 (8.2)	0.66	10(16)	9.5 (5.8)	1.63[e]
Esophageal	14(13)	8.9 (8.4)	1.05	14(11)	11.0 (8.9)	1.25	11(13)	15.8 (7.7)	2.06[e]	14(13)	10.6 (8.0)	1.33[e]	13(13)	9.0 (8.3)	1.08	15(13)	4.6 (8.7)	0.53[e]	14(14)	6.0 (7.8)	0.77
Testis	15(17)	7.2 (7.1)	1.01	16(16)	6.9 (7.4)	0.93	17(14)	6.2 (6.8)	0.91	18(17)	7.2 (5.9)	1.22	14(14)	8.9 (7.4)	1.20	12(17)	6.1 (7.2)	0.84	13(15)	6.6 (6.3)	1.06

PRCDA indicates Purchased/Referred Care Delivery Areas; AI/AN: American Indians/Alaska Natives; NHW: non-Hispanic white; RR: Rate Ratio.

[a] AI/AN race is reported by NPCR and SEER registries or through linkage with the IHS patient registration database. Includes only AI/AN of non-Hispanic origin. Source: Cancer registries in the Centers for Disease Control and Prevention's National Program of Cancer Registries (NPCR) and/or the National Cancer Institute's Surveillance, Epidemiology and End Results Program (SEER). Years of data and registries used: 1999-2016 (48 states): Containing at least one PRCDA county AK, AL, AZ, CA, CO, CT, FL, IA, ID, IN, KS, LA, MA, ME, MI, MN, MT, ND, NE, NM, NV, NY, NC, OK, OR, PA, RI, SC, TX, UT, WA, WI, WY; 2000-2016: SD; 2003-2016: MS. 1999-2016 States with no PRCDA Counties: DE, DC, GA, HI, IL, KY, MD, MO, NH, NJ, OH, TN, VA, VT, WV; 2000-2016: AR.

[b] Rank based on rates AI/AN rates overall. White rank in parenthesis

[c] Rates are per 100,000 persons and are age-adjusted to the 2000 U.S. standard population (19 age groups - Census P25-1130).
[d] RR are AI/AN versus White and are calculated in SEER*Stat prior to rounding of rates and may not equal RR calculated from rates presented in table.

Web Table 2. Leading Cancer Sites for American Indians/Alaska Natives[a] compared to Whites for the United States, Females, PRCDA Counties, US, 2012-2016

Site	Overall US			Northern Plains			Alaska			Southern Plains			Pacific Coast			East			Southwest		
	Rank[b], AI/AN (W)	AI/AN (W) Rate[c]	RR[d]	Rank[b], AI/AN (W)	AI/AN (W) Rate[c]	RR[d]	Rank[b], AI/AN (W)	AI/AN (W) Rate[c]	RR[d]	Rank[b], AI/AN (W)	AI/AN (W) Rate[c]	RR[d]	Rank[b], AI/AN (W)	AI/AN (W) Rate[c]	RR[d]	Rank[b], AI/AN (W)	AI/AN (W) Rate[c]	RR[d]	Rank[b], AI/AN (W)	AI/AN (W) Rate[c]	RR[d]
All Cancers		438.6 (431.8)	1.02		518.8 (419.6)	1.24[e]		554.0 (394.3)	1.41[e]		572.5 (414.1)	1.38[e]		451.3 (432.7)	1.04[e]		320.5 (458.4)	0.70[e]		305.8 (392.1)	0.78[e]
Female Breast	1(1)	112.1 (130.6)	0.86[e]	1(1)	123.3 (124.2)	0.99	1(1)	151.0 (121)	1.25[e]	1(1)	158.7 (119)	1.33[e]	1(1)	119.5 (133.5)	0.90[e]	1(1)	85.6 (137.1)	0.62[e]	1(1)	68.1 (121.5)	0.56[e]
Lung	2(2)	57.6 (54.5)	1.06[e]	2(2)	102.0 (53.3)	1.92[e]	3(2)	71.9 (47.8)	1.51[e]	2(2)	84.6 (56.6)	1.49[e]	2(2)	60.7 (51.5)	1.18[e]	2(2)	45.9 (60.9)	0.75[e]	6(2)	16.2 (47.6)	0.34[e]
Colorectal	3(3)	45.2 (32.4)	1.40[e]	3(3)	51.3 (33.0)	1.55[e]	2(3)	96.3 (32.6)	2.96[e]	3(3)	55.7 (35.3)	1.58[e]	3(3)	43.2 (32.0)	1.35[e]	3(3)	33.1 (32.9)	1.00	2(3)	29.6 (29.8)	0.99
Corpus and Uterus, NOS	4(4)	27.3 (26.4)	1.03	4(4)	25.2 (28.8)	0.88	6(4)	17.9 (25.0)	0.72[e]	4(4)	29.9 (21.9)	1.37[e]	4(5)	30.8 (26.7)	1.15[e]	4(4)	18.3 (27.6)	0.66[e]	3(5)	28.2 (22.5)	1.25[e]
Kidney	5(11)	20.3 (10.9)	1.87[e]	5(8)	23.5 (11.1)	2.12[e]	5(9)	21.3 (11.9)	1.79[e]	5(8)	27.7 (13.2)	2.10[e]	7(11)	16.5 (10.4)	1.59[e]	5(11)	14.6 (11.4)	1.28	5(11)	17.2 (9.3)	1.84[e]
Thyroid	6(6)	19.3 (22.4)	0.87[e]	7(6)	16.5 (19.8)	0.83	4(5)	23.4 (17.1)	1.37	6(5)	23.6 (20.1)	1.18[e]	5(6)	20.4 (20.2)	1.01	7(5)	11.0 (25.2)	0.44[e]	4(4)	18.5 (24.6)	0.75[e]
Non-Hodgkin Lymphoma	7(7)	15.5 (15.9)	0.98	6(7)	18.6 (16.2)	1.15	8(7)	14.4 (13.8)	1.04	7(7)	19.9 (15.1)	1.32[e]	6(7)	16.6 (15.7)	1.06	6(7)	11.2 (17.2)	0.65[e]	9(7)	11.7 (13.4)	0.87
Ovary	8(8)	12.2 (11.5)	1.07	8(11)	12.9 (10.7)	1.20	16(10)	7.6 (11.4)	0.67	9(9)	15.5 (11.6)	1.33[e]	11(8)	10.6 (11.9)	0.90	11(10)	8.1 (11.6)	0.70	7(8)	12.7 (11.0)	1.15
Pancreas	9(10)	12.0 (10.9)	1.1	9(9)	12.2 (10.9)	1.12	9(8)	13.5 (12.1)	1.12	8(11)	16.3 (10.2)	1.60[e]	9(9)	11.7 (10.9)	1.07	9(9)	8.5 (11.6)	0.74	10(9)	9.7 (9.9)	0.98
Cervical	10(14)	11.0 (6.7)	1.64[e]	10(14)	12.0 (6.3)	1.90[e]	11(13)	10.9 (6.8)	1.60	10(12)	13.8 (8.5)	1.63[e]	8(14)	13.8 (6.9)	2.00[e]	8(14)	8.9 (6.4)	1.38	13(13)	7.2 (6.3)	1.14
Liver	11(17)	10.7 (3.5)	3.03[e]	12(17)	10.5 (3.2)	3.27[e]	15(18)	7.7 (3.5)	2.17[e]	13(17)	10.3 (3.5)	2.93[e]	10(17)	11.2 (3.9)	2.85[e]	12(18)	7.4 (3.4)	2.17[e]	8(17)	12.1 (3.3)	3.63[e]
Leukemias	12(9)	9.8 (11.0)	0.89[e]	11(10)	10.8 (10.7)	1.00	14(11)	8.3 (9.8)	0.85	11(10)	13.4 (11.4)	1.18	12(10)	9.7 (10.7)	0.91	13(8)	6.5 (12.0)	0.54[e]	12(10)	7.9 (9.6)	0.82
Stomach	13(18)	7.7 (3.3)	2.32[e]	16(18)	5.9 (3.1)	1.92[e]	7(17)	14.5 (3.6)	4.07[e]	16(18)	6.8 (3.0)	2.27[e]	17(18)	6.1 (3.0)	2.03[e]	10(17)	8.2 (4.0)	2.05[e]	11(18)	8.5 (2.7)	3.16[e]
Melanomas of the Skin	14(5)	7.5 (23.4)	0.32[e]	18(5)	4.4 (21.5)	0.21[e]	13(6)	8.7 (16.5)	0.52[e]	12(6)	12.3 (17.4)	0.71[e]	13(4)	9.6 (27.1)	0.35[e]	16(6)	4.9 (23.3)	0.21[e]	16(6)	4.5 (20.4)	0.22[e]
Oropharyngeal	15(13)	6.7 (6.9)	0.97	13(13)	10.1 (6.7)	1.51[e]	10(15)	13.4 (5.1)	2.61[e]	17(14)	6.7 (7.1)	0.95	14(13)	8.4 (7)	1.20	14(13)	6.5 (7.4)	0.87	19(15)	2.6 (5.5)	0.47[e]

PRCDA indicates Purchased/Referred Care Delivery Areas; AI/AN: American Indians/Alaska Natives; NHW: non-Hispanic white; RR: Rate Ratio.

[a] AI/AN race is reported by NPCR and SEER registries or through linkage with the IHS patient registration database. Includes only AI/AN of non-Hispanic origin. Source: Cancer registries in the Centers for Disease Control and Prevention's National Program of Cancer Registries (NPCR) and/or the National Cancer Institute's Surveillance, Epidemiology and End Results Program (SEER). Years of data and registries used: 1999-2016 (48 states): Containing at least one PRCDA county AK, AL, AZ, CA, CO, CT, FL, IA, ID, IN, KS, LA, MA, ME, MI, MN, MT, ND, NE, NM, NV, NY, NC, OK, OR, PA, RI, SC, TX, UT, WA, WI, WY; 2000-2016: SD; 2003-2016: MS. 1999-2016 States with no PRCDA Counties: DE, DC, GA, HI, IL, KY, MD, MO, NH, NJ, OH, TN, VA, VT, WV; 2000-2016: AR.

[b] Rank based on rates AI/AN rates overall. White rank in parenthesis

[c] Rates are per 100,000 persons and are age-adjusted to the 2000 U.S. standard population (19 age groups - Census P25-1130).

[d] Rate ratios (RR) are AI/AN versus White and are calculated in SEER*Stat prior to rounding of rates and may not equal RR calculated from rates presented in table.

[e] Indicates RR is statistically significant ($p<0.05$).

Web Figure 1: Geographic Regions and Purchased/Referred Care Delivery Area[a] Counties by Region

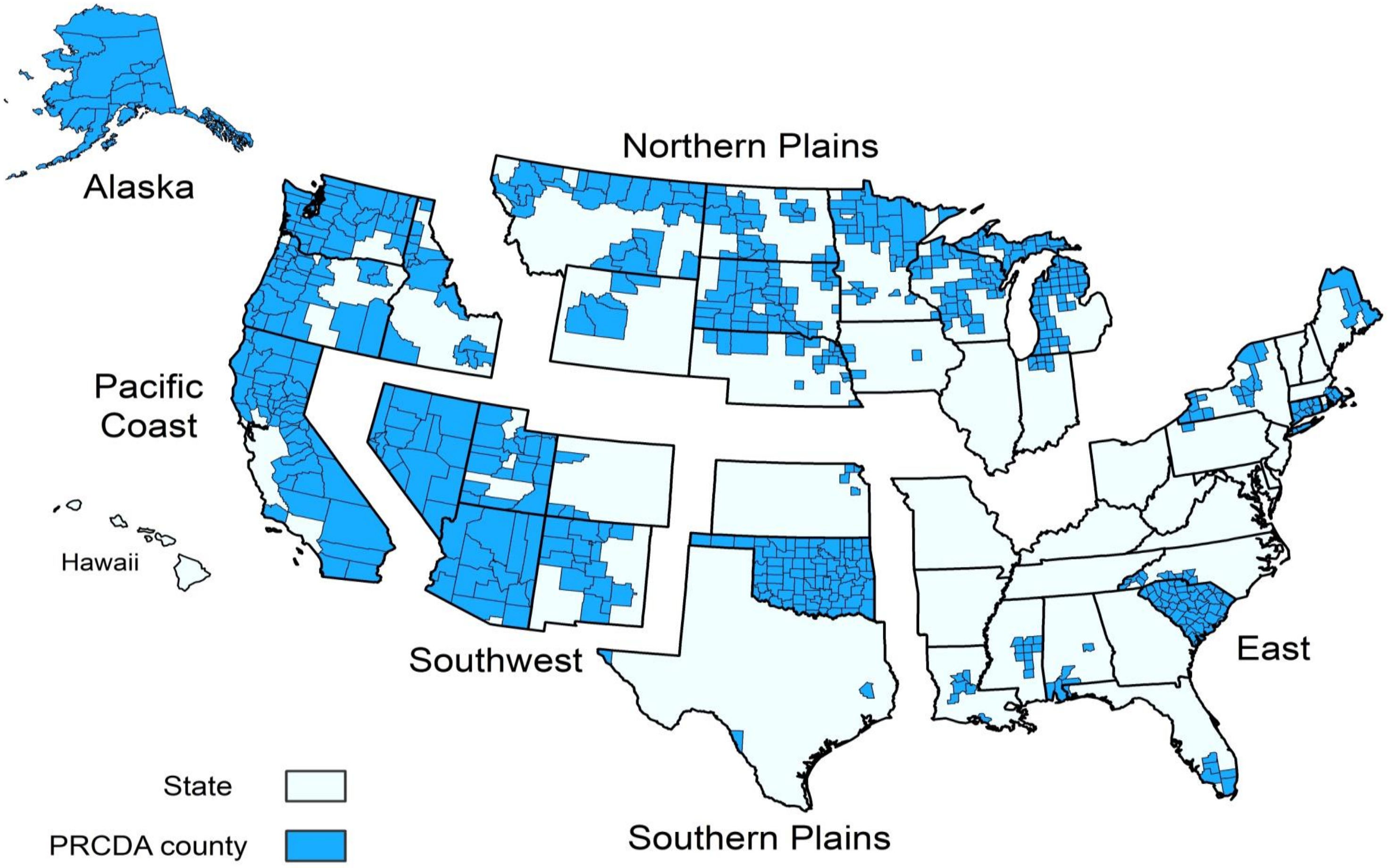

[a] Counties that contain federally recognized tribal lands or are adjacent to tribal lands. Only counties in blue are included in the analysis. Race classification for the AI/AN population is more accurate in these counties. Percent regional coverage of AI/AN in PRCDA counties to AI/AN in all counties: Northern Plains=54.2%; Alaska=100%; Southern Plains=56.5%; Southwest=83.8%; Pacific Coast=60.2%; East=16.4%; Total US=53.0%. Average Annual AI/AN population in PRCDA counties 2012-2016: Northern Plains= 339,493; Alaska=125,691; Southern Plains=426,098; Pacific Coast =628,284; East = 191,493; Southwest = 690,719; Total US = 2,401,778.

Source: Cancer registries in the Centers for Disease Control and Prevention's National Program of Cancer Registries (NPCR) and/or the National Cancer Institute's Surveillance, Epidemiology and End Results Program (SEER). Years of data and registries used: 1999-2016 (48 states): Containing at least one PRCDA county AK, AL, AZ, CA, CO, CT, FL, IA, ID, IN, KS, LA, MA, ME, MI, MN, MT, ND, NE, NM, NV, NY, NC, OK, OR, PA, RI, SC, TX, UT, WA, WI, WY; 2000-2016: SD; 2003-2016: MS. 1999-2016 States with no PRCDA Counties: DE, DC, GA, HI, IL, KY, MD, MO, NH, NJ, OH, TN, VA, VT, WV; 2000-2016: AR.
